# Supplementary material for: Proteomic Analysis of 3T3-L1 Adipocytes Treated with Insulin and TNF-α
Source: Proteomes. 2019 Oct 20;7(4):35. doi: 10.3390/proteomes7040035 (PMC6958341; doi:10.3390/proteomes7040035)
Supplement: Supplementary file 1 [file proteomes-07-00035-s001.zip › proteomes-594825-supplementary figure.pdf]

(A)

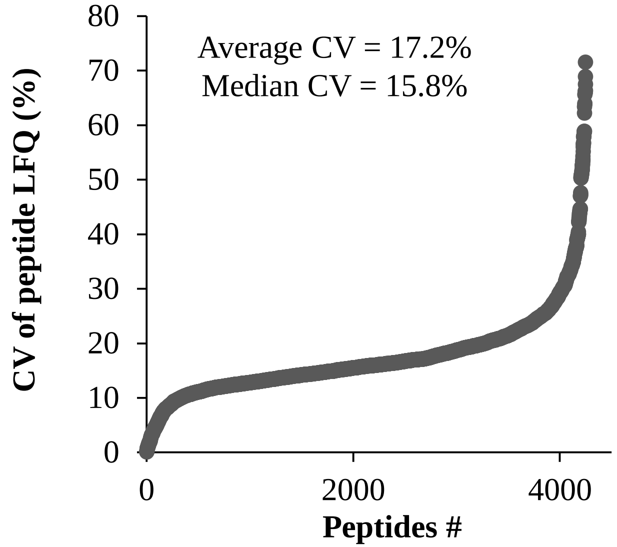

(B)

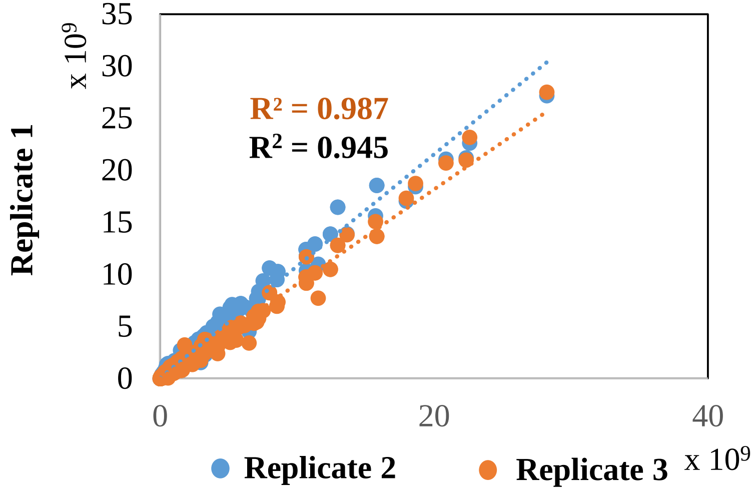

Fig. S1. LC-MS reproducibility. (A) Percentage CV (coefficient of variation), and (B) correlation coefficients of peptide intensities in 3 technical replicates.

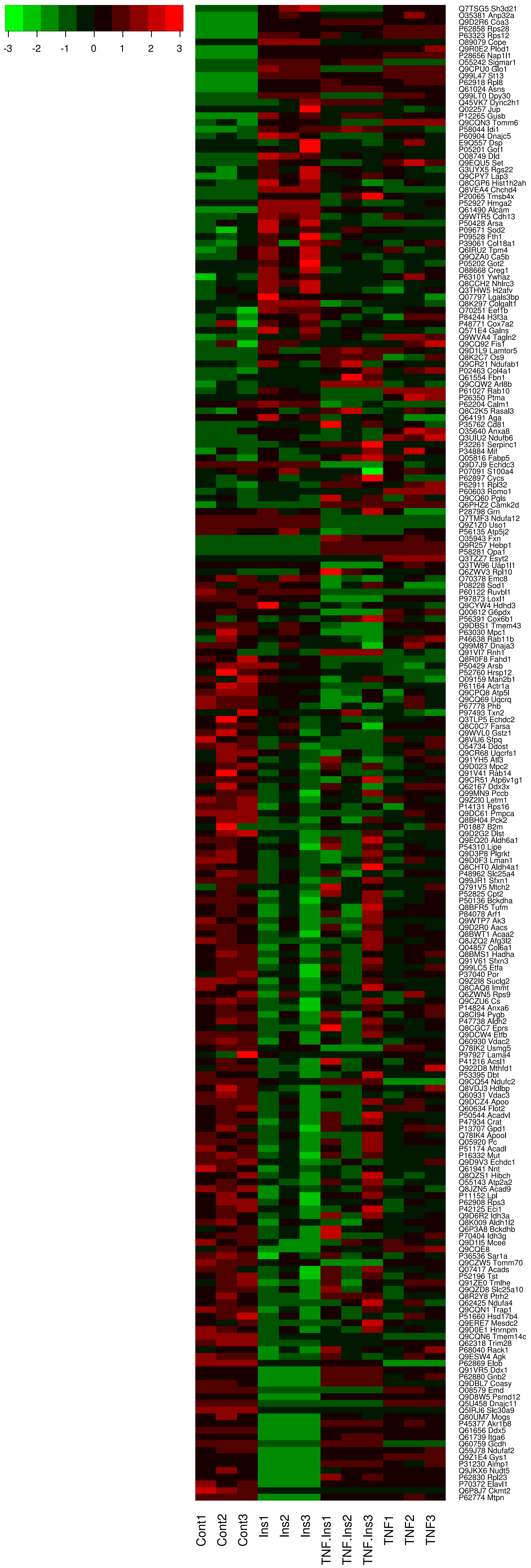
-3
-2
-1
0
1
2
3
Q7TSG5 Sh3d21
O35381 Anp32a
Q9D2R6 Coa3
P62858 Rps28
P63323 Rps12
O89079 Cope
Q9R0E2 Plod1
P28656 Nap1l1
O55242 Sigmar1
Q9CPU0 Glo1
Q99L47 St13
P62918 Rpl8
Q61024 Asns
Q99LT0 Dpy30
Q45VK7 Dync2h1
Q02257 Jup
P12265 Gusb
Q9CQN3 Tomm6
P58044 Idi1
P60904 Dnajc5
E9Q557 Dsp
P05201 Got1
O08749 Dld
Q9EQU5 Set
G3UYX5 Rgs22
Q9CPY7 Lap3
Q8CGP6 Hist1h2ah
Q8VEA4 Chchd4
P20065 Tmsb4x
P52927 Hmga2
Q61490 Alcam
Q9WTR5 Cdh13
P50428 Arsa
P09671 Sod2
P09528 Fth1
P39061 Col18a1
Q6IRU2 Tpm4
Q9QZA0 Ca5b
P05202 Got2
O88668 Creg1
P63101 Ywhaz
Q8CCH2 Nhlrc3
Q3THW5 H2afv
Q07797 Lgals3bp
Q8K297 Colgalt1
O70251 Eef1b
P84244 H3f3a
P48771 Cox7a2
Q571E4 Galns
Q9WVA4 Tagln2
Q9CQ92 Fis1
Q9D1L9 Lamtor5
Q8K2C7 Os9
Q9CR21 Ndufab1
P02463 Col4a1
Q61554 Fbn1
Q9CQW2 Arl8b
P61027 Rab10
P26350 Ptma
P62204 Calm1
Q8C2K5 Rasal3
Q64191 Aga
P35762 Cd81
O35640 Anxa8
Q3UIU2 Ndufb6
P32261 Serpinc1
P34884 Mif
Q05816 Fabp5
Q9D7J9 Echdc3
P07091 S100a4
P62897 Cycs
P62911 Rpl32
P60603 Romo1
Q9CQ60 Pgls
Q6PHZ2 Camk2d
P28798 Grn
Q7TMF3 Ndufa12
Q9Z1Z0 Uso1
P56135 Atp5j2
O35943 Fxn
Q9R257 Hebp1
P58281 Opa1
Q3TZZ7 Esyt2
Q3TW96 Uap1l1
Q6ZWV3 Rpl10
O70378 Emc8
P08228 Sod1
P60122 Ruvbl1
P97873 Loxl1
Q9CYW4 Hdhd3
Q00612 G6pdx
P56391 Cox6b1
Q9DBS1 Tmem43
P63030 Mpc1
P46638 Rab11b
Q99M87 Dnaja3
Q91VI7 Rnh1
Q8R0F8 Fahd1
P50429 Arsb
P52760 Hrsp12
O09159 Man2b1
P61164 Actr1a
Q9CPQ8 Atp5l
Q9CQ69 Uqcrq
P67778 Phb
P97493 Txn2
Q3TLP5 Echdc2
Q8C0C7 Farsa
Q9WVL0 Gstz1
Q8VIJ6 Sfpq
O54734 Ddost
Q9CR68 Uqcrfs1
Q91YH5 Atl3
Q9D023 Mpc2
Q91V41 Rab14
Q9CR51 Atp6v1g1
Q62167 Ddx3x
Q99MN9 Pccb
Q9Z2I0 Letm1
P14131 Rps16
Q9DC61 Pmpca
Q8BH04 Pck2
P01887 B2m
Q9D2G2 Dlst
Q9EQ20 Aldh6a1
P54310 Lipe
Q9D3P8 Plgrkt
Q9D0F3 Lman1
Q8CHT0 Aldh4a1
P48962 Slc25a4
Q99JR1 Sfxn1
Q791V5 Mtch2
P52825 Cpt2
P50136 Bckdha
Q8BFR5 Tufm
P84078 Arf1
Q9WTP7 Ak3
Q9D2R0 Aacs
Q8BWT1 Acaa2
Q8JZQ2 Afg3l2
Q04857 Col6a1
Q8BMS1 Hadha
Q91V61 Sfxn3
Q99LC5 Etfa
P37040 Por
Q9Z2I8 Suclg2
Q8CAQ8 Immt
Q6ZWN5 Rps9
Q9CZU6 Cs
P14824 Anxa6
Q8CI94 Pygb
P47738 Aldh2
Q8CGC7 Eprs
Q9DCW4 Etfb
Q60930 Vdac2
Q78IK2 Usmg5
P97927 Lama4
P41216 Acsl1
Q922D8 Mthfd1
P53395 Dbt
Q9CQ54 Ndufc2
Q8VDJ3 Hdlbp
Q60931 Vdac3
Q9DCZ4 Apoo
Q60634 Flot2
P50544 Acadvl
P47934 Crat
P13707 Gpd1
Q78IK4 Apool
Q05920 Pc
P51174 Acadl
P16332 Mut
Q9D9V3 Echdc1
Q61941 Nnt
Q8QZS1 Hibch
O55143 Atp2a2
Q8JZN5 Acad9
P11152 Lpl
P62908 Rps3
P42125 Eci1
Q9D6R2 Idh3a
Q8K009 Aldh1l2
Q6P3A8 Bckdhb
P70404 Idh3g
Q9D1I5 Mcee
Q9CQE8
P36536 Sar1a
Q9CZW5 Tomm70
Q07417 Acads
P52196 Tst
Q91ZE0 Tmlhe
Q9QZD8 Slc25a10
Q8R2Y8 Ptrh2
Q62425 Ndufa4
Q9CQN1 Trap1
P51660 Hsd17b4
Q9ERE7 Mesdc2
Q9D0E1 Hnrnpm
Q9CQN6 Tmem14c
Q62318 Trim28
P68040 Rack1
Q9ESW4 Agk
P62869 Elob
Q91VR5 Ddx1
P62880 Gnb2
Q9DBL7 Coasy
O08579 Emd
Q9D8W5 Psmd12
Q5U458 Dnajc11
Q5IRJ6 Slc30a9
Q80UM7 Mogs
P45377 Akr1b8
Q61656 Ddx5
Q61739 Itga6
Q60759 Gcdh
Q59J78 Ndufaf2
Q9Z1E4 Gys1
P31230 Aimp1
Q9JKX6 Nudt5
P62830 Rpl23
P70372 Elavl1
Q6P8J7 Ckmt2
P62774 Mtpn
Cont1
Cont2
Cont3
Ins1
Ins2
Ins3
TNF.Ins1
TNF.Ins2
TNF.Ins3
TNF1
TNF2
TNF3
